# Cytokine adsorption therapy in lymphoma-associated hemophagocytic lymphohistiocytosis and allogeneic stem cell transplantation

**DOI:** 10.1007/s10047-020-01244-2

**Published:** 2021-01-18

**Authors:** Jan-Gerd Rademacher, Gerald Wulf, Michael J. Koziolek, Michael Zeisberg, Manuel Wallbach

**Affiliations:** 1 Department of Nephrology and Rheumatology, University Medical Center Göttingen, Robert-Koch-Str. 40, 37075 Göttingen, Germany; 2 Department of Hematology and Medical Oncology, University Medical Center Göttingen, Göttingen, Germany

**Keywords:** Cytokine adsorption, Allogenic stem cell transplantation, Lymphoma, Hemophagocytic lymphohisticytosis, Inflammation

## Abstract

Lymphoma-associated Hemophagocytic lymphohistiocytosis (HLH) represents a severe complication of disease progression, mediated through cytokine release from the lymphoma cells. Cytokine adsorption may contribute as a supportive treatment to stabilize organ function by reduction of cytokine levels. So far, no experiences of cytokine adsorption and simultaneous stem cell transplantation were published. We report the case of a patient with aggressive lymphoma secondary to chronic lymphocytic leukemia with rapidly progressive HLH (Richter’s transformation) upon conditioning chemotherapy prior to allogeneic stem cell transplantation (ASCT). Continuous hemodiafiltration was initiated in the treatment of shock with acute renal failure, lactacidosis and need for high-dose catecholamine therapy, integrating an additional cytokine-adsorbing filter (CytoSorb**®**) to reduce cytokine levels. This was followed by scheduled allogenic stem cell transplantation. We observed a marked decrease in interleukin-6 plasma levels, associated with a reduced need for vasopressor therapy and organ function stabilization. Hematopoietic engraftment was present at day 14 post-ASCT, leading to disease-free discharge at day 100 post-transplantation. Cytokine adsorption may serve as a safe adjunct to HLH/sepsis treatment during allogeneic stem cell transplantation. Clinical studies are required to make future treatment recommendations.

## Introduction

Hemophagocytic lymphohistiocytosis (HLH) represents a severe and potentially lethal immune activation syndrome that can cause a sepsis-like condition (“cytokine storm”). Primary HLH mostly occurs in childhood while secondary forms may be triggered by viral infections, malignant diseases, in particular lymphomas, or several auto-immune diseases, where it is referred as macrophage activation syndrome [1]. Cytokine-adsorbing therapy constitutes a supportive extracorporal treatment option based on filter systems which remove cytokines via small polymer beads, overall reducing the imbalance between hyper-inflammation and anti-inflammatory messengers. It has been introduced for the treatment of septic shock with simultaneous indication for renal replacement therapy (RRT), and it has recently also been applied for intraoperative SIRS prophylaxis during heart–lung machine [2]. The indication may, however, also pertain to further conditions, such as HLH, MAS, liver failure, rhabdomyolysis or intoxications [3, 4]. Of note, cytokine adsorption also reduces the plasma levels of proteins measured for diagnostic purposes, such as procalcitonin (PCT) and liver transaminases [2]. The procedure has been employed successfully and safely applied at our center since 2018, mostly in combination with RRT in selected cases as an emergency procedure. To the best of our knowledge, the use of cytokine adsorption has not been reported in the course of stem cell transplantation.

## Case presentation

In the 57-year-old female patient, the diagnosis of HLH associated with diffuse large B-cell lymphoma (DLBCL) stage IS secondary to chronic lymphocytic leukemia (Richter transformation) was made five months before stem cell transplantation. The DLBCL had been detected as an isolated manifestation in the spleen, with complete resolution of HLH symptoms by splenectomy and subsequent treatment with 6 courses of R-CHOP immunochemotherapy. For remission consolidation, ASCT with cells of a matched unrelated donor was scheduled, and conditioning therapy with the FBC-12 protocol including lymphodepletion with anti-thymocyte globulin was initiated [5]. After completing conditioning therapy, the patient experienced shock symptoms with dyspnea necessitating intensive care. Due to concomitant acute renal failure, lactacidosis and need for high-dose catecholamine therapy, continuous hemodiafiltration with an additional cytokine-adsorbing filter (CytoSorb**®**) was initiated. RRT was performed as continuous hemodiafiltration (CHDF) with postdilution via *5008S CorDiax* dialysis machine that used high-flux filter system *FX CorDiax 60* (both Fresenius medical care). Dialysate flow was 800 ml/min and blood flow was between 200 and 250 ml/min. Heparin was administered for anticoagulation. CytoSorb**®** system was placed pre-filter. Following the manufacturers’ recommendations, CytoSorb**®** column and dialysis system including high-flux filter were replaced after a maximum of 24 h.

**Fig. 1:**
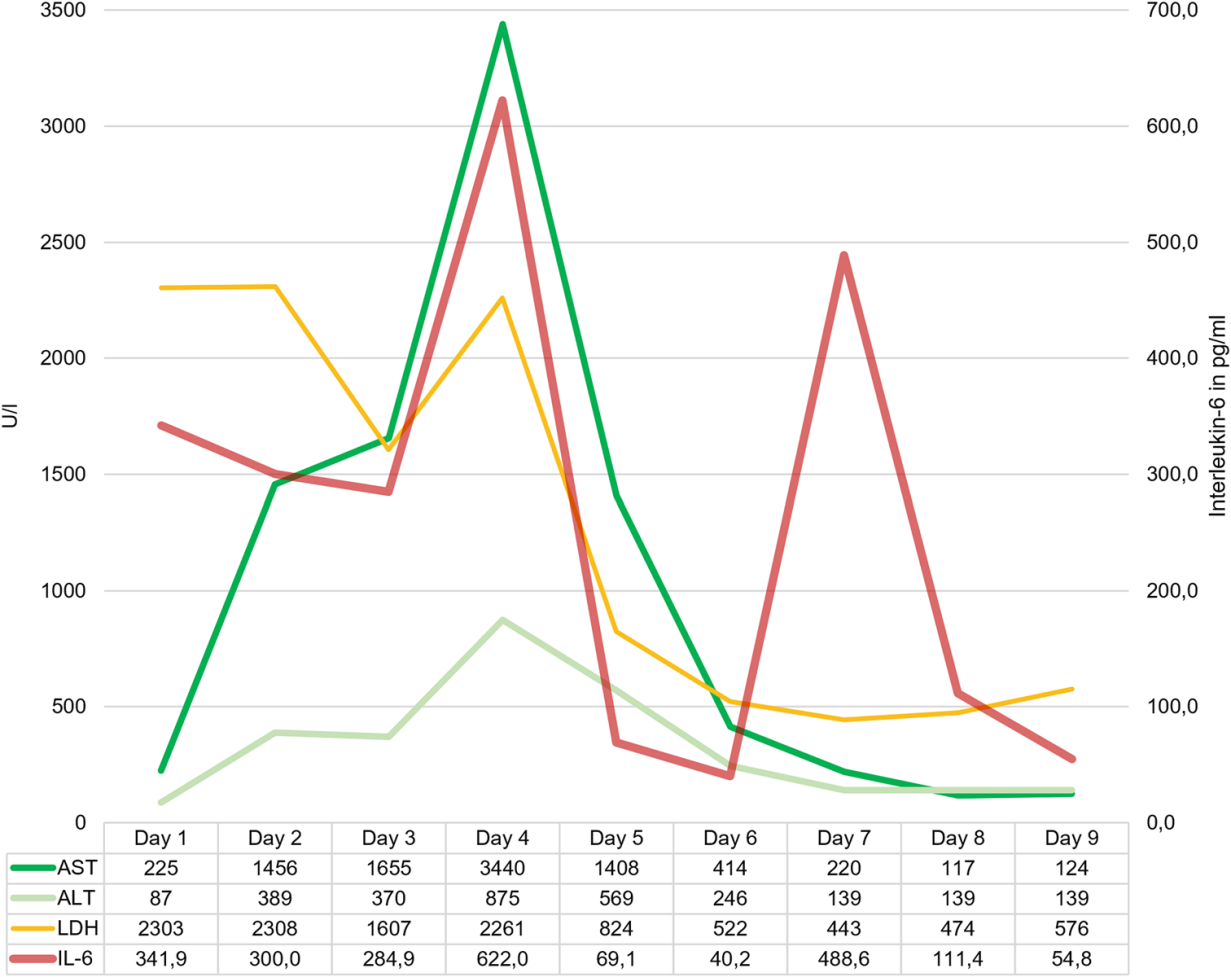
Progression of laboratory values presenting an acute liver failure/damage during the first 9 days after ICU admission. *ALT* alanine aminotransferase, *AST* aspartate aminotransferase, *IL-6* Interleukin 6, *LDH* lactate dehydrogenase

The ASCT with CD34 positive cells was performed according to plan at day 2 of hemodiafiltration with additional cytokine adsorption. C-reactive protein (CRP) and PCT were significantly elevated. In addition, acute liver failure occurred with a highly increase in transaminases (Fig. 1). Sonography of the abdomen showed formerly detected hepatosplenomegaly but regular portal vein and hepatic vein flow in Doppler. Multiple blood and urine cultures as well as virus PCR for EBV and CMV were negative. X-ray of the thorax was remarkable and showed no signs of infection. Continuous therapy with meropenem and vancomycin did not improve circulatory dysfunction or laboratory parameters of inflammation despite adequate drug monitoring. Ferritin was elevated with > 40.000 µg/l and an interleukin-6 (IL-6) level of 341.9 pg/ml could be measured before start of CytoSorb**®**.

In view of the massively increased ferritin, relapse of HLH with severe shock and multiorgan failure was to be assumed. HScore indicated a probability of > 98% for HLH, acknowledging the criteria may reliably pertain to the specific situation due to lymphoma and chemotherapy effects. By reasons of aplasia after chemotherapeutics, the great likelihood and missing therapeutic consequences a bone marrow aspiration was not performed.

**Fig. 2:**
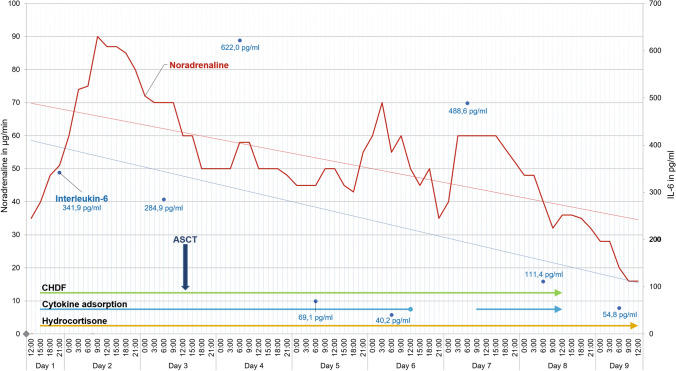
Catecholamine therapy (noradrenaline) and Interleukin-6 levels during the first 9 days under extracorporeal renal replacement therapy with the use of cytokine adsorption. ASCT was performed on day 3. The linear red and blue lines represent trends of the corresponding need for vasopressors and the IL-6 progression. *ASCT* allogenic stem cell transplantation, C*HDF* continuous hemodiafiltration, *IL-6* interleukin 6

Continuous hydrocortisone therapy and extracorporal RRT with supportive cytokine adsorption led to improved circulatory, decrease of lactate excess and stabilization of liver function. Figure 2 shows the course of required catecholamine dose and IL-6 concentrations during the first nine days. Ferritin decreased from > 40.000 µg/l to 2.878 µg/l within three weeks and even after extracorporal cytokine adsorption was omitted, indicating disease activity of HLH further declined. Leukocyte engraftment with donor chimerism of 99% on day 14 and 100% on day 100 after transplantation was measured. No signs of graft versus host diseases occurred until discharge from hospital. Renal dysfunction required intermitted RRT but recovered to an estimated glomerular filtration rate (eGFR) of 47 ml/min. The patient was transferred to the IMC ward after 20 days after transplantation and later discharged to rehabilitation.

## Discussion

Sepsis and the sepsis-like condition HLH show a high mortality in the intensive care setting. The *Simplified Acute Physiology Score* (SAPS) II provides an algorithm for calculating hospital mortality [6]. A propensity-score-based analysis of patients with septic shock highlighted that the additional usage of cytokine adsorption to RRT may lead to a reduction of SAPS II score and improved survival in the ICU setting [7]. In our patient, SAPS II predicted an estimated mortality of 88% (74 points) in the initial phase before onset of hemodialysis (Table 1). Further measurements of the score were not useful due to masking of urea and bilirubin by RRT with CytoSorb**®**. Recent reports outline that cytokine adsorption was primarily used in patients with high-predicted mortality, the observed outcome after cytokine removal, however, was distinctly lower [8].

**Table 1:** Predicted hospital mortality by Simplified Acute Physiology Score (SAPS) II

Variable	Points	% points
1. Urine output < 500 mL/day	11	14.9%
2. Hematologic malignancy	10	13.5%
3. P:F ratio 100–199	9	12.2%
4. Bilirubin ≥ 6 mg/dL	9	12.2%
5. Age 40–59 years	7	9.5%
6. Medical admission	6	8.1%
7. Bicarbonate < 15 mEq/L	6	8.1%
8. BUN 28–83 mg/dL	6	8.1%
9. Systolic BP 70–99 mmHg	5	6.8%
10. HR 120–159 bpm	4	5.4%
11. Sodium ≥ 145 mEq/L	1	1.4%
12. WBC 1.0–19.9 (10^3^/µL)	0	0%
13. Glasgow coma score 14–15	0	0%
14. Potassium 3.0–4.9 mEq/dL	0	0%
15. Temperature < 39 °C	0	0%
Total	74	100%
Predicted mortality	88%	

*BP* blood pressure, *BUN* blood urea nitrogen, *HR* heart rate *P:F* *ratio* arterial pO2 divided by the fraction of inspired oxygen (*Fi*O2), *WBC* white blood cell (count)

Currently, R-CHOP is the standard-of-care first-line chemotherapy regimen for DLBCL-HLH, whereas in refractory cases, intensive chemotherapy regimens are suggested [9]. Chemotherapy may not salvage these patients because of severe multiorgan failure at presentation leading to an overall mortality of > 90% [10]. In such cases, cytokine adsorption might bridge the patient until chemotherapy contributes to reduce HLH disease activity. The use of CytoSorb**®** leads to a decreasing demand for vasopressors and significant lactate clearance [11]. Besides, the stabilization of the circulatory system, ASCT was essential for the survival of our patient. In addition to other factors like matching genetic HLA markers, the success of an ASCT depends on migration of donor progenitor cells into the bone marrow and their consecutive growth. Certain profiles of chemokines and cytokines were detected and are thought to play a decisive role. Some cytokine patterns are associated with ASCT-induced complications and are prognostically relevant [12]. Although little is known about the connection between messenger profiles and success of stem cell transplantation, elevated levels of several cytokines, such as members of the IL-6 family before and after ASCT could increase the risk of Graft-versus-host-disease and transplant-related mortality [13]. While the exact etiology of hyper-inflammation in the case of this patient may be multifactorial, we presume that Anti-Thymocyte-Globulin (ATG) will have significantly contributed to cytokine release. ATG exerts its immunosuppressive effects through complement-dependent T-cell depletion, releasing cytokines from both host immune cells and residual tumor cells, which may result in macrophage activation [14, 15].

The spectrum of removed substances under CytoSorb**®** includes different endogenous messengers like chemokines [16]. Nevertheless, the messenger milieu required for ASCT does not appear to have been influenced to the detriment of the therapy. It is conceivable that these required cytokines were present in sufficient quantities despite the procedure. Moreover, some authors doubt that elimination from central blood flow will affect cytokine concentration in the capillary blood compartment, damaged/inflammated tissue or bone marrow significantly [17]. In view of the HLH relapse, IL-6 levels were only moderately elevated and the discontinuation of CytoSorb**®** on day six led to an immediate increase of IL-6, indicating persistent inflammation. There is a lack of randomized controlled trials and therefore it is not known whether cytokine adsorption is more effective at particularly high concentrations and which cytokines can predict its effectiveness [18]. Up to this point, it seems unclear whether cytokine-adsorbing therapy will establish itself as the icing on the cake in supportive sepsis treatment or as rescue strategy. In the case of the patient presented here, cytokine adsorption may have contributed to the mitigation of lymphoma-associated HLH under conditioning therapy, allowing successful ASCT.
